# An extremely heavy chlorine reservoir in the Moon: Insights from the apatite in lunar meteorites

**DOI:** 10.1038/s41598-019-42224-8

**Published:** 2019-04-05

**Authors:** Ying Wang, Weibiao Hsu, Yunbin Guan

**Affiliations:** 1 Key Laboratory of Planetary Sciences, Purple Mountain Observatory, Chinese Academy of Sciences, Nanjing, 210034 China; 2 The State Key Laboratory of Lunar and Planetary Science/Space Science Institute, Macau University of Science and Technology, Taipa, Macau, China; 3 CAS Center for Excellence in Comparative Planetology, Purple Mountain Observatory, Nanjing, 210034 China; 4 Division of Geological and Planetary Sciences, California Institute of Technology, Pasadena, CA 91125 USA

## Abstract

Chlorine, an extremely hydrophilic volatile element, provides important information regarding the origin of intrinsic volatiles in the Moon. Lunar apatite was found to have a wider spread of δ^37^Cl (from −1 to +40‰ versus standard mean ocean chloride) than most terrestrial and chondritic ones (0 ± 0.5‰). However, the provenance of the elevated lunar δ^37^Cl is still enigmatic. Here we report new isotopic data for H and Cl in apatite from three lunar meteorites and discuss possible mechanisms for Cl isotopic fractionation of the Moon. The apatite grain in Dhofar 458 has an average δ^37^Cl value of +76‰, indicative of an extremely heavy Cl reservoir in the Moon. Volatile loss associated with the Moon-forming Giant Impact and the formation of lunar magma ocean could account for the large Cl isotopic fractionation of the Moon. The observed H_2_O contents (220–5200 ppm), δD (−100 to +550‰) and δ^37^Cl values (+3.8 − +81.1‰) in lunar apatite could be understood if late accretion of hydrous components were added to the Moon after the fractionation of Cl isotopes. The heterogeneous distribution of lunar Cl isotopes is probably resulted from complex lunar formation and differentiation processes.

## Introduction

By virtue of recent analytical advances, considerable amounts of volatiles (H, C, F, S, and Cl) have been detected in lunar samples^1–6^, posing challenges to the favored Moon formation model^7^ in which most volatiles were thought to have been lost due to the Giant Impact between a Mars-sized body and the proto-Earth. On the other hand, a large variation of δ^37^Cl (from −1‰ to +40‰)^8–14^ was observed in the lunar samples. This appears to contradict the elevated volatile contents present in the lunar samples as the large Cl isotopic fractionation was ascribed to metal chlorides degassing from anhydrous magmas^8^. An alternative model argued that the residual melt of the lunar magma ocean (LMO), known as urKREEP (K, potassium; REE, rare earth elements; P, phosphorus), was the ^37^Cl-rich reservoir, with δ^37^Cl of ~+30‰^9,10^. Nevertheless, the correlation between high δ^37^Cl values and a KREEP component has been questioned^14–16^. A recent model^14^ proposed that the elevated δ^37^Cl values of apatite were partially inherited from vapor-phase metasomatism. However, the provenance of the ^37^Cl-rich gas is still enigmatic^12,14^. To better understand the origin and fractionation mechanism of lunar Cl, we conducted SIMS (secondary ion mass spectrometry) analyses on apatite [Ca_5_(PO_4_)_3_(F,Cl,OH)] from three lunar meteorites on the basis of detailed mineralogical and petrographic studies, and assess possible fractionation mechanisms and potential reservoirs of lunar Cl isotopes.

## Results

**Figure 1:**
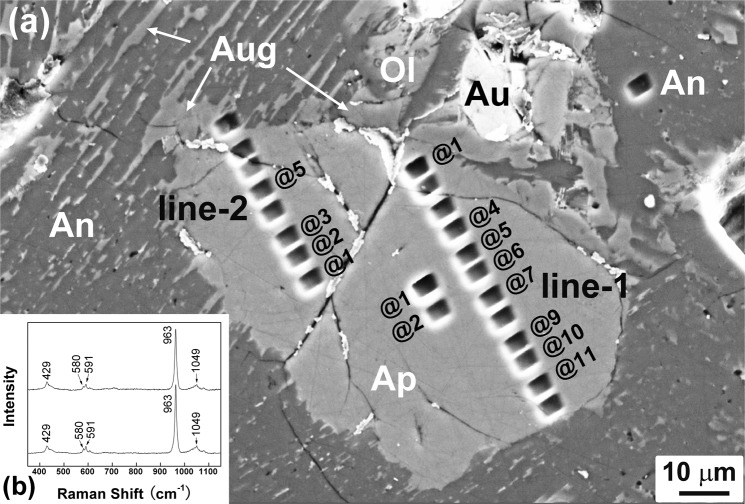
Petrographic and mineralogic characterization of the apatite grain in the feldspathic breccia Dhofar 458. (**a**) Back-scattered electron (BSE) image of the apatite grain, showing that it was subhedral and partially corroded by recrystallized anorthite-augite intergrowths. The rectangular pits are from NanoSIMS analyses. The spot numbers are labelled beside the pits. (**b**) Raman spectra of the apatite grain, exhibiting characteristic bands of F,Cl-apatite at 963, 429, 580, 591, and 1050 cm^−1^. Abbreviations: Ap, apatite; An, anorthite; Aug, augite; Ol, olivine.

In the mare basalt Miller Range (MIL) 05035 and the KREEP-assimilated gabbro Northwest Africa (NWA) 2977, subhedral to anhedral apatite (20–150 µm) commonly occurs in mesostasis along with other late-stage minerals (Fig. S1). Apatite contains REEs (0.09–1.98 wt%) that are positively correlated with its molar Fe/(Mg + Fe) and Cl/(Cl + F) ratios (Fig. S2), which is in agreement with the incompatibility of REEs and Cl over F during magmatic evolution. In the feldspathic breccia Dhofar 458, one subhedral apatite grain (60 × 100 µm, Fig. 1a) partially corroded by anorthite-augite intergrowths was found in an anorthositic troctolite clast (Fig. S1c). The apatite grain contains only trace amounts of REEs (Ce_2_O_3_, 0.08 wt%; Nd_2_O_3_, 0.05; Y_2_O_3_, 0.04; Table S1). All apatite grains studied here are F-rich (1.9–3.4 wt%; Table 1), indicative of a magmatic origin^12,17^.

**Table 1:** Chemical (ppm) and isotopic (‰) compositions of volatile elements in apatite from lunar meteorites.

	δ^37^Cl	2σ	H_2_O	2σ	F (wt%)	2σ	Cl	2σ	δD	2σ	S	2σ
**MIL 05035, 31**												
Ap-1@1	5.1	3.1	3500	210	2.37	0.23	6500	130	450	30	320	80
@2	5.5	3.1	4000	230	2.28	0.22	6000	130			310	80
@3	5.9	3.1	3900	230	2.27	0.22	6500	130			150	80
@4	6.3	3.2	4100	240	2.17	0.22	6000	130			130	80
@5	3.8	3.1	4800	270	2.31	0.22	5800	130			180	80
@6	5.6	3.3	4200	240	2.11	0.22	4100	110			110	80
Ap-2@1	5.5	2.9	5100	280	1.94	0.21	14300	240	370	40	210	80
@2	6.3	3.0	4800	270	1.95	0.21	13900	230	370	30	200	80
@3	6.6	2.9	4700	260	2.18	0.22	13700	230			180	80
@4	4.5	3.0	5200	280	2.18	0.22	12600	210			140	80
@5	4.1	3.0	4500	250	1.93	0.21	10500	180			120	80
Ap-3@1	5.8	3.0	2200	170	2.23	0.22	7200	140			50	80
Ap-5@1	7.8	3.3	3700	220	2.32	0.23	5100	120	410	30	120	80
**NWA 2977, PMO-0244**												
Ap-1@1	8.5	3.5	2600	60	3.44	0.18	200	80			70	100
Ap-3@1	21.8	3.0	1900	60	3.44	0.18	800	90	−120	20	60	100
@2	23.1	2.9	1300	50	3.35	0.18	1100	90	−100	30	60	100
@3	20.6	3.1	1400	50	3.44	0.18	800	90			60	100
**NWA 2977, PMO-0095**												
Ap-3b@1	23.7	1.9	1300	130								
@2	26.2	1.8	1100	120								
Ap-6@1	18.3	1.9	1700	140								
@2	16.7	1.9	2000	150								
**Dhofar 458, PMO-1026**												
Ap @1	74.3	1.9	260	40	2.83	0.17	9700	170	460	90	30	100
@2	67.2	2.0	250	40	2.82	0.17	10000	170	550	90	20	100
line-1 @1	78.9	2.0	250	40	3.02	0.17	10800	180			10	100
@4	76.9	2.0	220	40	3.00	0.17	10900	190			10	100
@5	76.1	2.0	260	40	2.82	0.17	10000	170			80	100
@6	72.1	2.1	220	40	2.73	0.16	10000	170			20	100
@7	75.8	2.1	220	40	2.96	0.17	10900	190			10	100
@9	77.2	2.2	230	40	2.93	0.17	10600	180			10	100
@10	72.0	2.1	220	40	2.75	0.16	9100	160			20	100
@11	76.7	2.2	240	40	2.89	0.17	10400	180			20	100
line-2 @1	78.3	2.1	250	40	2.70	0.16	10400	180			40	100
@2	78.8	2.0	250	40	2.77	0.16	11100	190			130	100
@3	81.1	2.0	230	40	2.69	0.16	10800	180			30	100
@5	80.8	2.1	260	40	2.76	0.16	10200	180			50	100

**Figure 2:**
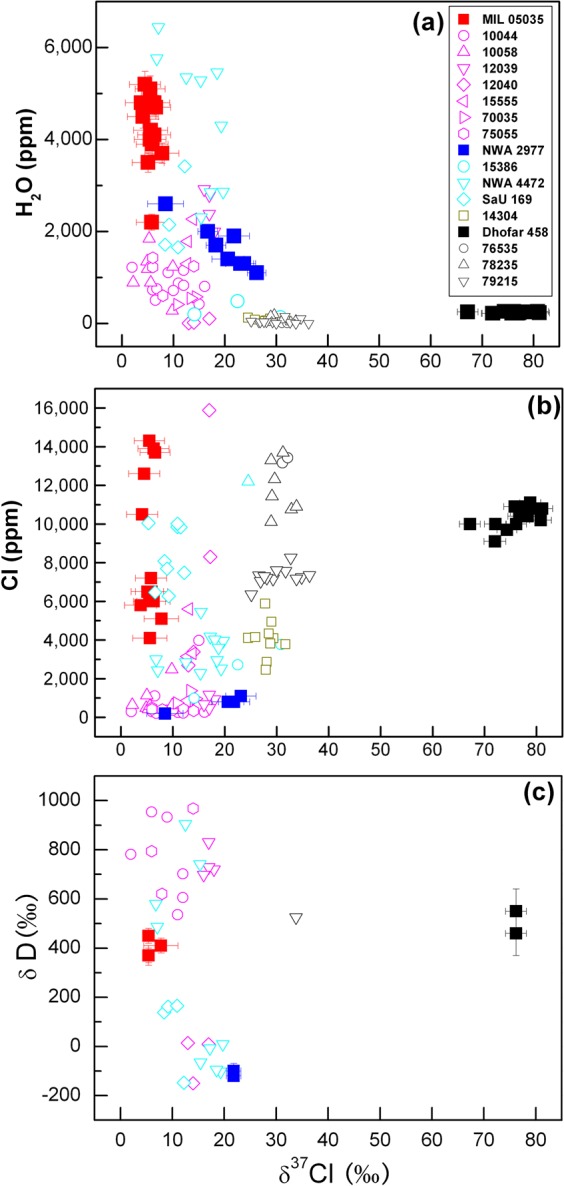
Plots of (**a**) H_2_O content, (**b**) Cl content, and (**c**) δD versus δ^37^Cl for lunar apatite from MIL 05035, NWA 2977, and Dhofar 458. Data from the literature (hollow symbols)^8–12^ are also plotted for comparison. Red and magenta symbols represent mare basalts; blue and cyan symbols are KREEP-bearing basalts; black and grey symbols are highland rocks; and 14304 is a very high-K basalt. No apparent correlation can be observed among δ^37^Cl, δD, and Cl content.

Eight apatite grains from MIL 05035 and NWA 2977 yielded δ^37^Cl values from +3.8 ± 3.1 (2σ) to +26.2 ± 1.8‰ and H_2_O contents from 1100 ± 120 to 5200 ± 280 ppm (Table 1, Fig. 2), which are comparable with previous reports^2–4,8–14^. Cl isotope compositions are homogeneous within individual grains. In Dhofar 458, the only apatite grain found has extremely high δ^37^Cl values of +67.2 ± 2.0 to +81.1 ± 2.0‰ and a relatively low H_2_O content averaging 240 ± 40 ppm (Table 1). The mean δD values of the apatite vary from −110 ± 30‰ in NWA 2977 to +400 ± 80‰ in MIL 05035, and to +510 ± 130‰ in Dhofar 458 (Table 1). No apparent correlation is observed among δ^37^Cl, Cl content, and δD in the apatite (Fig. 2).

## Discussion

Most terrestrial rocks have a very restricted δ^37^Cl variation (0 ± 0.5‰)^18^. The uniformity of terrestrial δ^37^Cl has been attributed to HCl degassing from hydrous magmas, in which only negligible Cl fractionation occurred^8^. The elevated lunar δ^37^Cl and its wide dispersion therefore have been ascribed to metal chlorides degassing from anhydrous magmas, and the lunar interior was suggested to be extremely depleted in hydrogen (lower than Earth by a factor of ~10^4^ to 10^5^)^8^. However, studies of volatiles in lunar volcanic glasses, melt inclusions, and anorthosites have found robust evidence indicating that the lunar interior contains, at least partially, considerable amounts of water^1,5,6^. Our analyses of lunar apatite yield high H_2_O contents (220–5200 ppm) consistent with previous data^2–4,9–12,14^, but it is not trivial to determine the H_2_O content of the parental magma because the simple Nernst partition is not applicable to F, Cl, and OH in apatite^19^.

Because in some mare and highland samples, the δ^37^Cl values of apatite are positively correlated with Cl contents and bulk compositions of trace incompatible elements, it was inferred that the Cl isotopes were mixtures from two distinct reservoirs: one is the lunar mantle with δ^37^Cl of ~0‰ and the other is urKREEP with δ^37^Cl of ~+30‰^9,10^. The ^37^Cl enrichment of lunar samples was ascribed to the contamination of KREEP components which is enriched in heavy Cl and incompatible elements^9,10^. However, this relationship seems to be only valid within some low-Ti and KREEP-rich basalts^14–16^. The δ^37^Cl values of apatite from Apollo 14 high-Al samples do not show any correlation with bulk La/Lu ratios^14^, and the results of high-Ti basalts are not apparently correlated^9,10,16^. This might be understandable because the high-Al mare basalts could have assimilated a mixture of KREEP and granite^20^, and the high-Ti basalts might have sampled their trace elements from a reservoir distinct from urKREEP^21^. Our results show that although the apatite from NWA 2977 and Dhofar 458 has a rough positive correlation between the Cl content and δ^37^Cl value, no correlation has been observed in the apatite of MIL 05035 (Fig. 2b). More importantly, in spite of the extremely high δ^37^Cl values (+67.2 to +81.1‰), the apatite in Dhofar 458 has only trace amounts of REEs (Table S1), and the whole-rock incompatible element abundances are relatively low (e.g., 0.37 µg/g of Th, 3.06 µg/g of La, and 1.22 µg/g of Sm)^22^. It is apparent that the high δ^37^Cl of Dhofar 458 is decoupled from contamination of KREEP components. The highly fractionated Cl isotopes in Dhofar 458 might have originated from a ^37^Cl-enriched reservoir distinct from the previously suggested urKREEP^9,10^.

In order to understand the fractionation mechanism of Cl isotopes in the Moon, especially the extremely high δ^37^Cl of the apatite in Dhofar 458, all relevant physiochemical processes are considered: (i) solar nebula condensation; (ii) vaporization, re-condensation, and accretion during the hypothesized giant impact; (iii) massive degassing of the LMO; (iv) partial melting of mantle sources and crystallization of minerals; (v) evaporation at fumaroles; (vi) magma degassing; (vii) shock-induced melting, evaporation, and condensation; and (viii) surface alterations.

Initially, the pristine solar nebula had a δ^37^Cl of ~−5 to −3‰^23^. The HCl hydration that followed caused a small degree of Cl isotope fractionation (3–6‰)^23^. Thus, most chondritic materials would eventually have a δ^37^Cl around 0‰^18^. Processes (ii) and (iii) would greatly enhance the fractionation of Cl isotopes and will be discussed later. Partial melting and crystallization at igneous temperatures would hardly fractionate Cl isotope composition^24^.

Fumarole processes in terrestrial system could produce Cl isotopic fractionation up to 16‰ via a distillation-like mechanism, which requires >99% evaporated HCl (g) to recondense to Cl^−^_aq_ in the liquid water along the fumarole conduit^25^. However, for lunar fumarole processes, the distillation-like mechanism could hardly proceed due to the extremely low pressure on the near-surface of the Moon^26,27^.

Anhydrous magmatic degassing of Cl-bearing species would lead to kinetic fractionation of Cl isotopes, i.e. ^35^Cl escapes more readily than ^37^Cl^8^. During this process, Cl concentration of the residual melt decreases gradually and δ^37^Cl value increases. Therefore, apatite grains crystallized from the magma are expected to show a negative correlation between δ^37^Cl and the Cl content. To the contrary, we found that the apatite with lower Cl content has lighter Cl isotopes in NWA 2977 (Table 1). Apatite grains in MIL 05035 have a large variation in Cl contents (4100–14300 ppm) but display almost identical δ^37^Cl (from +3.8 ± 3.1‰ to +7.8 ± 3.3‰). It might be possible that magmatic degassing had caused the Cl fractionation before apatite saturation. In this case, the Cl isotopes incorporated into apatite would not have to be correlated with the Cl content. However, one must be aware that an elevated δ^37^Cl could only be produced through extreme Cl loss^8^. Under the condition of Rayleigh distillation, +80‰ fractionation of Cl isotopes would require >99% volatilization of Cl in the magma (Fig. S3). However, most apatite grains analyzed here and reported in literatures^8–12,14^ contain considerable amounts of Cl. If apatite had crystallized from a magma with a high δ^37^Cl, a mechanism must be invoked to concentrate the highly fractionated Cl isotopes, e.g., the reservoir of urKREEP^9,10^. Therefore, we argue that magmatic degassing alone could not account for the large Cl isotope fractionation in the lunar samples.

The δ^37^Cl values are not correlated with the extent of shock metamorphism among lunar samples. The mare basalts MIL 05035 and NWA 2977 experienced a similar degree of shock metamorphism (refer to SI), but apatite in MIL 05035 has much lower δ^37^Cl than that of NWA 2977 (Table 1). For the Mg-suite highland rocks Apollo 76535 and 78235, the former is unshocked^28^ and the latter had been heavily shocked to ~50 GPa^29^, but their apatites have nearly identical δ^37^Cl values (~+30‰)^10^. Therefore, shock metamorphism would not be responsible for the lunar Cl fractionation as illustrated further in the later section.

Surface alteration, including micrometeorite bombardment and solar wind sputtering, could cause evaporation and isotopic fractionation of some volatile elements^30,31^ (e.g., S, C, and K). Studies of Cl isotopes of lunar regolith have been limited so far. Available data suggests no correlation between δ^37^Cl and the maturity of the soil^8^. Proton bombardment experiment simulating solar wind implantation yields no Cl isotopic fractionation^8^. Therefore, surface alteration might have played an insignificant role, but would definitely not be a major process in the Cl isotopic fractionation for most lunar samples.

Considering all of the scenarios discussed above, it is still not apparent what process is responsible for the fractionation of lunar Cl isotope, but there is a hint that the elevated lunar Cl isotope could be endogenous, developed during the Moon-forming processes. We are left with a scenario of the Giant Impact event in which the large Cl isotope fractionation could be induced during the energetic impact and massive evaporation of the LMO^9^. As previously suggested^32^, the formation of Moon involves the presence of a LMO which accreted from the vapor phases generated by the Giant Impact on the proto-Earth. Large fractions of volatile elements (e.g., K, Zn, and Cl) were lost at extremely high temperatures (up to ~1800 °C)^32^, and the vapor would enrich ^37^Cl through preferentially losing ^35^Cl to space (Rayleigh distillation). A similar scenario was suggested to account for the K and Zn isotope fractionations in the Moon^31,33,34^. The LMO remained in a liquidus state for about 10 to 200 Myr after the Giant Impact event, followed by the formation of mantle cumulates and an early anorthosite crust a few kilometers thick. These early formed lunar rocks are expected to carry a high ^37^Cl signature (^37^Cl/^35^Cl: ~0.344) as observed in Dhofar 458. It is possible that urKREEP, the last dreg of LMO crystallization, had concentrated volatiles and elevated δ^37^Cl values due to metal chloride evaporation, but our data is consistent with more than one highly fractionated Cl reservoir in the Moon. For the future work, it is highly desired to analyze Cl isotope compositions from primitive lunar anorthosites.

The reported ages for Dhofar 458 and its paired stone Dhofar 026 range from 0.57 ± 0.01 Ga to 3.43 ± 0.01 Ga^35–37^. Taken at face value, this stone is not a primitive lunar crust rock. However, these ages do not represent the formation time of Dhofar 458 but record a strong shock impact event at ~3.4 Ga^35^.

Because Dhofar 458 had been severely shocked and experienced extensive partial melting^38^, it is vital to ascertain the shock influence on the Cl isotopes of apatite. Lines of petrographic evidence support that the apatite was not crystallized from a shock-induced melt, but rather a relict grain. It has a large grain size (60 × 100 µm) and subhedral morphology (Fig. 1a) distinct from the skeletal crystals and fine-grained intergrowths crystallized from the shock-induced melt^38^. The edge of the apatite grain had been partially corroded by the recrystallized plagioclase-augite intergrowth (Fig. 1a), indicating that the formation of apatite preceded the formation of the intergrowth. If apatite was crystallized from a shock-induced melt, it should crystallize after the augite-plagioclase intergrowth. Therefore, the apatite grain is a relict grain partially corroded by shock-induced melt. It has been noted that under elevated temperatures (1100–2300 °C) and pressures (10–15 GPa), apatite could lose its volatile components and transform into a high-pressure polymorph tuite [γ-Ca_3_(PO_4_)_2_] through solid-state phase transition, decomposition, or crystallization from a shock-induced melt^39^. However, mineralogical evidence demonstrates that the shock-induced devolatilization and thus Cl isotope fractionation is minimal for the apatite in Dhofar 458. First, Raman spectra of the apatite exhibit characteristic bands of F,Cl-apatite at 963, 429, 580, 591, and 1050 cm^−1^ (Fig. 1c); the sharp narrow peaks indicate almost intact crystallinity. Second, this apatite grain has abundant F and Cl with a virtually ideal structural formula (Table S1). If the apatite experienced considerable solid-state devolatilization, lattice vacancy and decrease of F and Cl contents should be observed. Actually, the shock pressure and post-shock temperature were unevenly distributed within Dhofar 458, and the shock-induced melt had cooled and crystallized rapidly^40^. Therefore, we conclude that the shock process had only led to negligible halogen loss for the apatite grain in Dhofar 458. The high δ^37^Cl of the relict apatite was most likely inherited from its parental melt. There might be a reservoir in the Moon with δ^37^Cl value above +80‰, which was induced from the Moon-forming processes.

Experimental results have shown that under low pressure (<1 bar) and low oxygen fugacity, H-rich vapors evaporate rapidly and efficiently from the magma^26^. We speculate that immediately after the birth of the LMO, H-rich vapors, such as H_2_, HCl, HF, and H_2_S, might have lost from the surface or sub-surface of the LMO. After the fractionation of the Cl isotopes, hydrous components would be imported, which is compatible with the model that volatiles were accreted to the Moon as a “late veneer” during the crystallization period (~10–200 Myr) of the LMO^4,41–43^.

Our results show that the H_2_O and Cl contents and δ^37^Cl and δD values of lunar apatite exhibit distinct inter- and/or intra-sample variations (Table 1), which are basically compatible with previous reports^1–14^ that water and Cl are heterogeneously distributed in the Moon. The mechanism for the heterogeneity of lunar volatiles is not fully understood^44,45^. The variations of water content and D/H ratio of lunar samples have been ascribed to, at least, magmatic degassing, regolith assimilation, and delivery of impactors^4,44–46^. Nevertheless, our knowledge on the Cl signature of asteroid and comet impactors is limited. Available data from several carbonaceous chondrites exhibit a narrow range of δ^37^Cl (from −2.1 to +1.2‰)^18^. Surface alterations are believed to have negligible effect on the lunar Cl isotope ratio^8^. Therefore, although contributions from impactors cannot be ruled out completely, it is conceivable that the heterogeneity of lunar Cl is mainly due to endogenous mechanism, i.e. the complex lunar formation and differentiation processes^47^ including the secondary metasomatism suggested by Potts *et al*.^14^

## Methods

Three polished thin sections of MIL 05035, 31 (loaned from MWG), Dhofar 458 (PMO-1026), and NWA 2977 (PMO-0095), and one polished specimen of NWA 2977 (PMO-0244) mounted in indium were studied. Apatite grains were identified with a Hitachi S-3400N scanning electron microscope (SEM) equipped with an Oxford X-Max 20 energy dispersive spectroscope, housed at Purple Mountain Observatory (PMO).

The chemical compositions of the minerals were measured with an electron microprobe (JEOL JXA-8230) at PMO. The accelerating voltage was 15 kV. The beam current was 20 nA. To minimize the halogen migration, F and Cl were measured first in each channel, and the integral times were halved to 10 s/5 s at the peak and background positions. Both synthetic and natural mineral standards were used, and ZAF and XPP (for apatite only) corrections were applied. The Durango apatite was analyzed as a secondary standard during analytical sessions. After measuring the major and minor elements, the REEs (Ce, Pr, and Nd detected by qualitative analysis) and Y in apatite were analyzed separately, using a beam current of 40 nA, beam diameters of 3–8 μm, and a collecting time of 30 s/15 s. The concentrations of the major and minor elements obtained were input as set compositions for accurate data correction. The detection limit of the REEs and Y was 80–160 ppm.

The Raman spectra of the apatite were obtained at PMO with a Thermo Fisher DXR micro-Raman spectrometer, using a 532 nm laser, 2 mW laser power, and 25 μm pinhole aperture. The estimated spot size was 0.7 μm and the spectral resolution was 2 cm^−1^.

Volatile contents and Cl isotope compositions of the apatite were measured with the Cameca NanoSIMS 50 L ion microprobe at Caltech. A primary Cs^+^ beam of 8 keV and ~12 pA was used, with a normal-incidence electron gun for sample charging compensation. Ion images of ^12^C^−^, ^16^O^1^H^−^, and ^35^Cl^−^ were used to locate grain boundaries and to monitor any cracks or hot spots in grains before and after each analysis. Samples were pre-sputtered for ~5 min. Secondary ions of ^12^C^−^, ^16^O^1^H^−^, ^18^O^−^, ^19^F^−^, ^32^S^−^, ^35^Cl^−^, and ^37^Cl^−^ were simultaneously detected with electron multipliers (EMs). The raster size of the primary beam was 3 × 3 µm^2^, but only secondary ions from the center area (1.5 × 1.5 µm^2^) were collected in order to avoid edge contamination. The mass resolving powers (MRPs) were ~10,000, sufficient to separate all significant interferences from the masses of interest, such as ^17^O^−^ from ^16^O^1^H^−^, ^31^P^1^H^−^ from ^32^S^−^, and ^19^F^16^O^−^ from ^35^Cl^−^. Secondary ions were integrated for 200–2000 cycles (1 sec/cycle) based on volatile abundances. The mass peaks were auto-centered after every 100–200 cycles.

Calibrations for OH (reported as H_2_O equivalent), F, Cl, and S contents were based on natural apatites from Durango (Ap-3), Colorado (Ap-5), and Russia (Ap-18). Apatite standards and a San Carlos olivine grain were polished and embedded in an indium disk. The chemical compositions of the apatite standards were analyzed by Francis M. McCubbin at the University of New Mexico, Albuquerque, using an electron microprobe and H manometry^3^ (Table S3). At the beginning of an analytical session, apatite standards were analyzed and calibration curves were obtained (Fig. S4). To reduce instrumental background, all samples were kept in the sample chamber overnight before analysis. The chamber vacuum during analysis was (1–8) × 10^–10^ torr. Instrumental background was removed by measuring San Carlos olivine for apatite standards and adjacent plagioclase for lunar apatite. The reported uncertainties include both counting statistics and errors for the calibration curves. The δ^37^Cl values were corrected to the Durango apatite (+0.4‰).

After NanoSIMS analysis, all sections were re-polished and re-carbon-coated for measurement of H isotopes using the Cameca 7f-Geo ion microprobe at Caltech. The primary ^16^O^−^ beam of 3–7 nA and −13 keV was focused to a diameter of 10–15 µm. The sample was pre-sputtered over 10 × 10 µm areas for ~5 min, and then the beam raster size was reduced to 1 × 1 µm area for data collection. Secondary ions, ^1^H^+^ and ^2^H^+^, were collected in peak-jumping mode with an EM. A 90% e-gate was applied to avoid edge contamination. The MRP was 1500. All samples were kept in the sample chamber overnight before analysis. Liquid nitrogen and a Ti-sublimation pump were used to maintain the chamber vacuum below 8 × 10^−10^ torr. The instrumental background was estimated from the San Carlos olivine and lunar plagioclase and was determined to contribute to the ^1^H^+^ count rate by <1% if the apatite had ~5000 ppm water. For the Durango apatite (~300 ppm water), the background contribution was ~10%. The δD values of apatite were standardized using the Durango apatite (δD = −120 ± 5‰). Due to the short cosmic ray exposure ages of our samples (<12 Ma, Supplementary Information) and the relatively high H_2_O content of apatite, spallogenic D only accounts for negligible proportions of the deuterium (<1%). Therefore, no corrections for spallogenic D were applied here.

In an independent session, we measured the water contents of the apatite in a different thin section of NWA 2977 (PMO-0095) using the Cameca 7f-Geo ion microprobe. The results were similar to those obtained from the section (PMO-0244) embedded in indium. Working conditions were as follows: primary Cs^+^ beam of 4 nA and 10 keV, impact energy of 19 keV, and an electron-gun was applied. Samples were pre-sputtered over 25 × 25 µm areas for 120 s. The primary beam, with a diameter of ~20 µm, was rastered over 10 × 10 µm areas for data collection. ^16^O^1^H^−^ and ^31^P^−^ ions were detected with an EM, using ^31^P^−^ as a reference mass. The MRP was 6000, which is high enough to separate ^17^O^−^ from ^16^O^1^H^−^. An 80% e-gate was used. The calibration curve and background corrections were made using the same procedure as those from the NanoSIMS analysis.

After each working session, all craters on the sample surface were examined carefully with an SEM. Only data from clean apatite regions were adopted.

## Supplementary information

Supplementary Information

## Data Availability

All data analyzed during this study are included in this published article (and its Supplementary Information files).
