# Supplementary material for: An extremely heavy chlorine reservoir in the Moon: Insights from the apatite in lunar meteorites
Source: Sci Rep. 2019 Apr 5;9:5727. doi: 10.1038/s41598-019-42224-8 (PMC6450942; doi:10.1038/s41598-019-42224-8)
Supplement: Supplementary file 1 — Supplementary Information [file 41598_2019_42224_MOESM1_ESM.docx]

An extremely heavy chlorine reservoir in the Moon: Insights from the apatite in lunar meteorites

Ying Wang^1,2,3^, Weibiao Hsu^2,3*^, and Yunbin Guan^4^

1. Purple Mountain Observatory, Chinese Academy of Sciences, Nanjing 210034, China.
2. The State Key Laboratory of Lunar and Planetary Science/Space Science Institute, Macau University of Science and Technology, Taipa, Macau
3. CAS Center for Excellence in Comparative Planetology, Purple Mountain Observatory, Nanjing 210034, China.
4. Division of Geological and Planetary Sciences, California Institute of Technology, Pasadena, CA 91125, USA.

^*^e-mail: [wbxu@pmo.ac.cn](mailto:y_wang@pmo.ac.cn)

**Supplementary Information**

1. **Sample descriptions**

**1.1. MIL 05035**

MIL 05035 is an unbrecciated very low-Ti to low-Ti basalt composed of 54-62 vol% pyroxene, 25-36% plagioclase, and accessory minerals including quartz, ilmenite, spinel, apatite, and troilite^1,2^. It has a low bulk content of incompatible elements, suggesting derivation from a mantle source region depleted in KREEP component^1-2^. MIL 05035 has high affinities with Asuka 881757, Yamato 793169, and the basaltic component in MET 01210 (YAMM basalt), which might be source crater-paired^1-3^.

MIL 05035 had been shocked severely^1-3^. All plagioclase was transformed to maskelynite, and silica has planar deformation features (PDFs). Pyroxene was fractured, dislocated, and exhibits undulatory extinction, PDFs, and possible mosaicism. Pyroxferroite was breakdown due to shock-induced heating and troilite was melted into fractures. According to Stöffler et al.^4^, its shock stage reached S5, and the estimated peak pressure was ~10-80 Ga^1-2^.

Crystallization age of MIL 05035 had been determined with Ar-Ar, Sm-Nd, Rb-Sr, and Pb-Pb methods, which yielded consistent ages between 3.80-3.90 Ga^5-7^.

MIL 05035 was minimally weathered. Black and shiny fusion crust covers 95% of its exterior, and there is no rusting interior. The bulk contents of Rb (0.49 ppm), Sr (105 ppm), and Ba (25.8 ppm) are as low as un-weathered rocks^1^. MIL 05035 was well-shielded from solar wind^3^, and has a comic-ray exposure (CRE) age of 1-3 Ma^5^.

**1.2. NWA 2977**

NWA 2977 is a cumulate olivine gabbro composed of 41 vol% olivine, 39% orthopyroxene, 12% augite, 7% plagioclase, and minor oxide, phosphate, and K,Ba-feldspar^8^. It is paired with the olivine gabbro part of NWA 773. Both of them were formed in a hypabyssal setting and had close affinity to KREEP basalts^8-9^.

NWA 2977 had experienced shock metamorphism to various extents (S3-S6)^8^. Shock-induced melt veins and pockets are present. Plagioclase was partly transformed into maskelynite, olivine into ringwoodite, and chromite into its high-pressure polymorph. The post-shock pressure was heterogeneously distributed in the rock, estimated from 15 to 90 GPa^4,8^.

The crystallization age of NWA 2977 had been measured with Ar-Ar, Sm-Nd, Rb-Sr, and Pb-Pb methods, which varies from 2.77 ± 0.04 Ga (Ar-Ar) to 3.29 ± 0.11 Ga (Rb-Sr)^8,10-11^.

NWA 2977 is a fusion-encrusted stone and experienced minimal terrestrial weathering. It has low contents of Sr (45 ppm), Ba (134 ppm), Br (0.2 ppm), and As (0.06 ppm)^8-9^. The REE patterns of olivine, pyroxene, and whole rock did not show any Ce anomaly^8^. There was little alteration of FeNi metal and sulfide^8^. The sparse carbonate deposited in fractures can be easily avoided during micro-beam analyses. NWA 2977 was well-shielded from solar wind and has a CRE age of ~12 Ma^11-12^.

**1.3. Dhofar 458**

Dhofar 458 is a feldspathic breccia composed primarily of olivine-plagioclase and pyroxene-plagioclase intergrowths with some plagioclase fragments^13^. It is paired with Dhofar 026^14^. Different opinions, a granulitic breccia^15-16^ and impact melt rock^13-14^, exist regarding its petrogenesis.

The apatite-bearing clast (Fig. S1c) in our section consists of 66.0 vol% plagioclase, 23.5% olivine, 8.2% clinopyroxene, and 2.3% apatite, which is similar to the apatite-rich ferroan anorthositic troctolite clast in ALHA 81005^17^.

Dhofar 458 had been subjected to an intensive shock. On the basis of plagioclase occurrence, the equilibrium shock pressure and post-shock temperature were estimated to be 30-45 GPa and ~900 ºC^15^. But the melting of mafic minerals would indicate shock pressures >75 GPa and temperature ~1200 ºC^15^. And the decomposition of zircon may indicate localized pressure of ~60 GPa and temperature >1700 ºC^13^.

The U-Pb age of Dhofar 458 is 3434 ± 15 Ma, which recorded the time of an impact event^13^.

No apparent evidence for terrestrial weathering has been found in our section, although the paired stone NWA 026 was partially weathered^14-15^. The CRE age for Dhofar 026 is 10.9 ± 2.2 Ma^18^.

**References for Supplementary Information**

1. Joy, K. H., Crawford, I. A., Anand, M., Greenwood, R. C., Franchi, I. A. & Russell, S. S. The petrology and geochemistry of Miller Range 05035: A new lunar gabbroic meteorite. *Geochim. Cosmochim. Acta* **72**, 3822–3844 (2008).
2. Liu, Y., Floss, C., Day, J. M. D., Hill, E. & Taylor, L. A. Petrogenesis of lunar mare basalt meteorite Miller Range 05035. *Meteorit. Planet. Sci.* **44**, 261–284 (2009).
3. Arai, T., Ray Hawke, B., Giguere, T. A., Misawa, K., Miyamoto, M. & Kojima, H. Antarctic lunar meteorites Yamato-793169, Asuka-881757, MIL 05035, and MET 01210 (YAMM): Launch pairing and possible cryptomare origin. *Geochim. Cosmochim. Acta* **74**, 2231–2248 (2010).
4. Stöffler, D., Keil, K. & Scott, E. R. D. Shock metamorphism of ordinary chondrites. *Geochim. Cosmochim. Acta* **55**, 3845–3867 (1991).
5. Fernandes, V. A., Burgess, R. & Morris, A. ^40^Ar-^39^Ar age determinations of lunar basalt meteorites Asuka 881757, Yamato 793169, Miller Range 05035, La Paz Icefield 02205, Northwest Africa 479, and basaltic breccia Elephant Moraine 96008. *Meteorit. Planet. Sci.* **44**, 805–821 (2009).
6. Zhang, A., Hsu, W., Li, Q., Liu, Y., Jiang, Y. & Tang, G. SIMS Pb/Pb dating of Zr-rich minerals in lunar meteorites Miller Range 05035 and LaPaz Icefield 02224: Implications for the petrogenesis of mare basalt. *Sci China Earth Sci* **53**, 327–334 (2010).
7. Nyquist, L. E., Shih, C.-Y. & Reese, Y. D. Sm-Nd and Rb-Sr ages for MIL 05035: Implications for surface and mantle sources. *38^th^ Lunar Planet. Sci. Conf.* abstr. 1702 (2007).
8. Zhang, A., Hsu, W., Floss, C., Li, X., Li, Q., Liu, Y. & Taylor, L. A. Petrogenesis of lunar meteorite Northwest Africa 2977: Constraints from in situ microprobe results. *Meteorit. Planet. Sci.* **45**, 1929–1947 (2010).
9. Jolliff, B. L., Korotev, R. L., Zeigler, R. A. & Floss, C. Northwest Africa 773: Lunar mare breccia with a shallow-formed olivine-cumulate component, inferred very-low-Ti (VLT) heritage, and a KREEP connection. *Geochim. Cosmochim. Acta* **67**, 4857–4879 (2003).
10. Nyquist, L. E., Shih, C.-Y., Reese, Y. D. & Irving, A. J. Sm-Nd and Rb-Sr ages for Northwest Africa 2977, a young lunar gabbro from the PKT. *72^nd^ Annual Meteoritical Soc. Meeting* abstr. 5347 (2009).
11. Burgess, R., Fernandes, V. A., Irving, A. J. & Bunch, T. E. Ar-Ar ages of NWA 2977 and NWA 3160 – Lunar meteorites paired with NWA 773. *38^th^ Lunar Planet. Sci. Conf.* abstr. 1603 (2007).
12. Lorenzetti, S., Busemann, H. & Eugster, O. Regolith history of lunar meteorites. *Meteorit. Planet. Sci.* **40**, 315–327 (2005).
13. Zhang, A., Hsu, W., Li, X., Ming, H., Li, Q., Liu, Y. & Tang, G. Impact melting of lunar meteorite Dhofar 458: Evidence from polycrystalline texture and decomposition of zircon. *Meteorit. Planet. Sci.* **46**, 103–115 (2011).
14. Warren, P. H., Ulff-Møller, F. & Kallemeyn, G. W. “New” lunar meteorites: Impact melt and regolith breccias and large-scale heterogeneities of the upper lunar crust. *Meteorit. Planet. Sci.* **40**, 989–1014 (2005).
15. Cohen, B. A., James, O. B., Taylor, L. A., Nazarov, M. A. & Barsukova, L. D. Lunar highland meteorite Dhofar 026 and Apollo sample 15418: Two strongly shocked, partially melted, granulitic breccias. *Meteorit. Planet. Sci.* **39**, 1419–1447 (2004).
16. James, O. B., Cohen, B. A., Taylor, L. A. & Nazarov, M. A. Comment on: "New" lunar meteorites: Impact melt and regolith breccias and large-scale heterogeneities of the upper lunar crust, by P. H. Warren, F. Ulff-Moller, and G. W. Kallemeyn. *Meteorit. Planet. Sci.* **42**, 1029–1032 (2007).
17. Goodrich, C. A., Taylor, G. J. & Keil, K. An apatite-rich, ferroan, mafic lithology from lunar meteorite ALHA 81005. *Proc. 15^th^* *Lunar Planet. Sci. Conf.*, *J. Geophy. Res.* **90**, C405–C414 (1985).
18. Fernandes, V. A., Anand, M., Burgess, R. & Taylor, L. A. Ar-Ar studies of Dhofar clast-rich feldspathic highland meteorites: 025, 026, 280, 303 (abstract). *35^th^ Lunar Planet. Sci. Conf.* abstr. 1514 (2004).
19. McCubbin, F. M., Steele, A., Hauri, E. H., Nekvasil, H, Yamashita, S. & Hemley, R. J. Nominally hydrous magmatism on the Moon. *Proc. Natl. Acad. Sci. USA* **107**, 11223–11228 (2010).


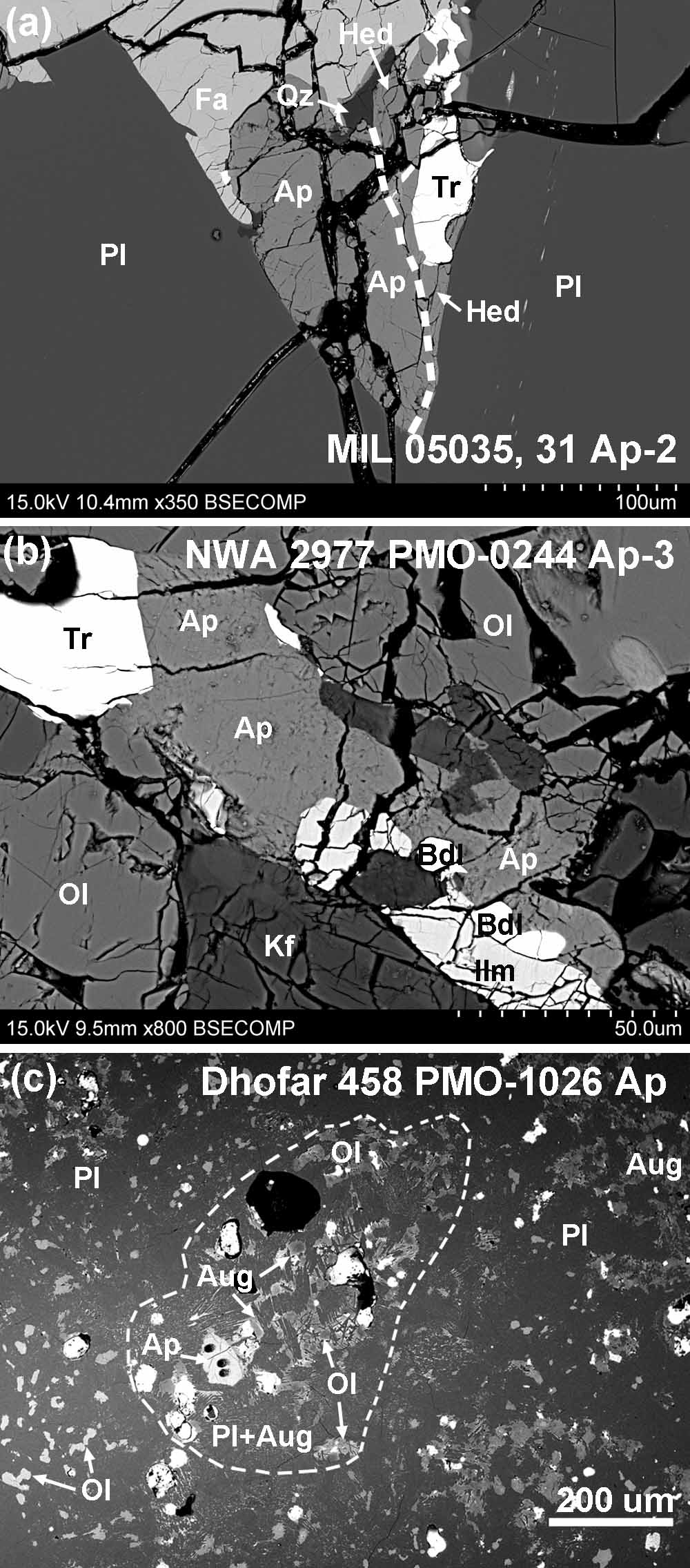


**Fig. S1. Back-scattered electron (BSE) images of lunar apatite.** The apatite (Ap) grains in MIL 05035 (a) and NWA 2977 (b) usually occur in the mesostasis with other late stage minerals like troilite (Tr), hedenbergite (Hed), quartz (Qz), fayalite (Fa), baddeleyite (Bdl), and K-feldspar (Kf). In Dhofar 458, the apatite grain occurs in an anorthositic troctolite clast (circled by the dashed line) composed mainly of 66 vol% plagioclase (Pl), 24% olivine (Ol), and 8% augite (Aug). Around the clast are pyroxene-plagioclase and olivine-plagioclase intergrowths. The pits on the apatite grain are from δD analysis with a Cameca 7f-geo ion microprobe. The lightest spots are mostly Au particles that were not removed after Au-coating.

**Fig. S2. Plots of total content of REEs (including Ce, Pr, Nd, and Y) versus molar ratios of Cl/(Cl+F) and Fe/(Fe+Mg) of the apatite from NWA 2977 and MIL 05035.** Each symbol represents a single apatite grain. The REE contents of apatite are positively correlated with its molar Cl/(Cl+F) and Fe/(Mg+Fe) ratios, conforming to the incompatibility of REEs and Cl over F during magmatic evolution.


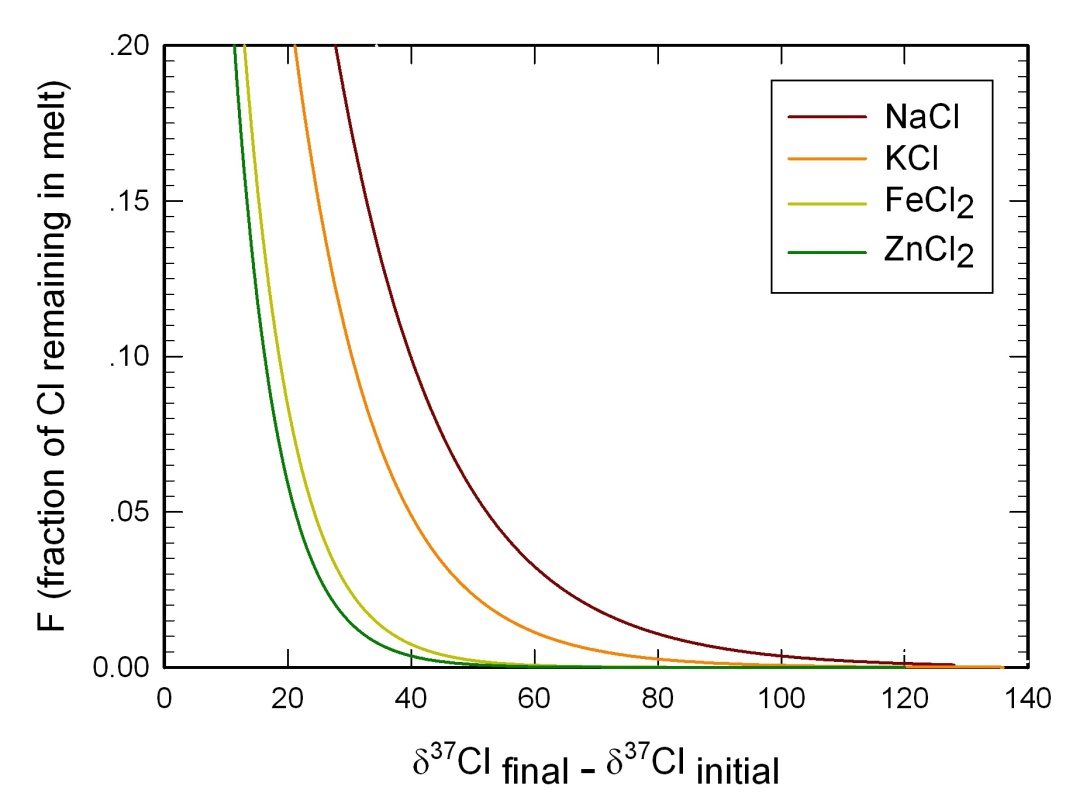


**Fig. S3 Fractionation trajectories of Cl isotopes of lunar magmas, varying with the proceeding of magma degassing.** A process of Rayleigh distillation is assumed for calculation, where δ_final_ = δ_initial_ + [(1000 + δ_initial_) (F ^(α-1)^ - 1)]. The values of fractionation factor (α) are 0.983 for NaCl, 0.987 for KCl, 0.992 for FeCl_2_, and 0.993 for ZnCl_2_**.** More than 99% Cl in the magma has to be lost in order to yield +80‰ fractionation of δ^37^Cl.

**Fig. S4. Calibration curves for H_2_O (a), F (b), Cl (c) and S (d) contents in lunar apatite, based on analyses of terrestrial apatite standards Ap-3, -5, and -18 (Table A3).** Uncertainties are 1σ.

**Table S1. Average and representative mineral chemistry (wt%, EPMA) of apatite from lunar meteorites.**

|  | MIL 05035 | |  | NWA 2977 | |  | Dhofar 458 | | | | | | | |
| --- | --- | --- | --- | --- | --- | --- | --- | --- | --- | --- | --- | --- | --- | --- |
|  | N=13 | s.d. |  | N=16 | s.d. |  | N=29 | s.d. | #18^*^ | #19^*^ | #20^*^ | #21^*^ | #22^*^ | #23^*^ |
| SiO_2_ | 0.83 | 0.26 |  | 0.20 | 0.10 |  | 1.40 | 1.16 | 0.37 | 0.26 | 0.29 | 0.39 | 1.95 | 1.43 |
| FeO | 0.96 | 0.42 |  | 0.73 | 0.42 |  | 0.93 | 0.37 | 0.58 | 0.59 | 0.57 | 0.53 | 0.93 | 1.04 |
| MgO | 0.02 | 0.02 |  | 0.16 | 0.04 |  | 0.76 | 0.28 | 0.64 | 0.58 | 0.60 | 0.63 | 0.93 | 0.66 |
| CaO | 53.3 | 0.56 |  | 54.1 | 0.64 |  | 52.8 | 0.98 | 53.3 | 53.4 | 53.3 | 53.4 | 52.3 | 53.1 |
| Na_2_O | bd |  |  | bd |  |  | bd |  | 0.02 | 0.03 | 0.03 | bd | 0.03 | bd |
| P_2_O_5_ | 41.0 | 0.62 |  | 41.9 | 0.56 |  | 41.0 | 1.19 | 43.2 | 42.1 | 42.1 | 42.0 | 40.5 | 40.6 |
| Ce_2_O_3_ | 0.38 | 0.25 |  | 0.15 | 0.06 |  | 0.08 | 0.02 |  |  | 0.13 |  |  |  |
| Pr_2_O_3_ | 0.09 | 0.05 |  | 0.03 | 0.02 |  | bd |  |  |  | 0 |  |  |  |
| Nd_2_O_3_ | 0.27 | 0.12 |  | 0.09 | 0.05 |  | 0.05 | 0.03 |  |  | 0.09 |  |  |  |
| Y_2_O_3_ | 0.42 | 0.09 |  | 0.11 | 0.08 |  | 0.04 | 0.02 |  |  | 0.07 |  |  |  |
| F | 1.69 | 0.21 |  | 2.67 | 0.22 |  | 2.49 | 0.30 | 2.55 | 2.88 | 3.04 | 2.78 | 2.66 | 2.23 |
| Cl | 0.70 | 0.23 |  | 0.21 | 0.10 |  | 1.09 | 0.10 | 1.14 | 1.17 | 1.19 | 1.10 | 1.14 | 1.12 |
| -O=F | -0.71 |  |  | -1.13 |  |  | -1.05 |  | -1.07 | -1.21 | -1.28 | -1.17 | -1.12 | -0.94 |
| -O=Cl | -0.16 |  |  | -0.05 |  |  | -0.25 |  | -0.26 | -0.26 | -0.27 | -0.25 | -0.26 | -0.25 |
| Total: | 98.8 |  |  | 99.2 |  |  | 99.4 |  | 100.5 | 99.5 | 99.9 | 99.4 | 99.0 | 99.0 |
| Structural formulae based on 25 oxygen ions | | | | | | | | | | | | | | |
| n Si | 0.14 |  |  | 0.03 |  |  | 0.24 |  | 0.06 | 0.04 | 0.05 | 0.06 | 0.33 | 0.24 |
| n Fe | 0.14 |  |  | 0.10 |  |  | 0.13 |  | 0.08 | 0.08 | 0.08 | 0.07 | 0.13 | 0.15 |
| n Mg | 0.00 |  |  | 0.04 |  |  | 0.19 |  | 0.16 | 0.15 | 0.15 | 0.16 | 0.24 | 0.17 |
| n Ca | 9.73 |  |  | 9.80 |  |  | 9.55 |  | 9.47 | 9.65 | 9.61 | 9.65 | 9.48 | 9.64 |
| n Na | 0.00 |  |  | 0.00 |  |  | 0.00 |  | 0.01 | 0.01 | 0.01 | 0.00 | 0.01 | 0.00 |
| n P | 5.92 |  |  | 5.99 |  |  | 5.86 |  | 6.07 | 6.01 | 6.01 | 5.99 | 5.80 | 5.82 |
| n Ce | 0.02 |  |  | 0.01 |  |  | 0.01 |  |  |  | 0.01 |  |  |  |
| n Pr | 0.01 |  |  | 0.00 |  |  | 0.00 |  |  |  | 0.00 |  |  |  |
| n Nd | 0.02 |  |  | 0.01 |  |  | 0.00 |  |  |  | 0.01 |  |  |  |
| n Y | 0.04 |  |  | 0.01 |  |  | 0.00 |  |  |  | 0.01 |  |  |  |
| n F | 0.91 |  |  | 1.43 |  |  | 1.33 |  | 1.34 | 1.54 | 1.62 | 1.48 | 1.42 | 1.20 |
| n Cl | 0.20 |  |  | 0.06 |  |  | 0.31 |  | 0.32 | 0.33 | 0.34 | 0.31 | 0.33 | 0.32 |
| n OH | 0.88 |  |  | 0.51 |  |  | 0.36 |  | 0.34 | 0.13 | 0.04 | 0.20 | 0.25 | 0.48 |
| Cations | 16.02 |  |  | 15.99 |  |  | 15.98 |  | 15.84 | 15.95 | 15.93 | 15.94 | 15.98 | 16.02 |

s.d. = standard deviation; bd = below detection limit.

*Data points that constitute the F profile in Fig. 1.

**Table S2. Average and representative mineral chemistry (wt%, EPMA) of pyroxene, olivine, and anorthite from the apatite-bearing clast in Dhofar 458.**

|  | Pyroxene | | | | |  | Olivine | | | | | |  | Anorthite | | | | | |
| --- | --- | --- | --- | --- | --- | --- | --- | --- | --- | --- | --- | --- | --- | --- | --- | --- | --- | --- | --- |
|  | #11 | #23 | #26 | N=7 | s.d. |  | #31 | #32 | #33 | #34 | N=14 | s.d. |  | #2 | #3 | #10 | #11 | N=14 | s.d |
| SiO_2_ | 49.8 | 48.6 | 49.3 | 49.4 | 0.66 |  | 39.0 | 34.9 | 36.9 | 37.1 | 38.0 | 1.40 |  | 45.4 | 43.7 | 44.1 | 44.3 | 44.1 | 0.51 |
| Al_2_O_3_ | 4.74 | 4.04 | 5.04 | 4.61 | 0.48 |  | 0.14 | 0.05 | 1.30 | 0.05 | 0.18 | 0.32 |  | 34.5 | 35.7 | 33.8 | 35.5 | 35.2 | 0.53 |
| TiO_2_ | 1.08 | 1.76 | 1.31 | 1.39 | 0.23 |  | 0.04 | 0.08 | 0.02 | 0.06 | 0.04 | 0.02 |  | - | - | - | - | - |  |
| Cr_2_O_3_ | 1.15 | 0.63 | 0.83 | 0.96 | 0.24 |  | 0.28 | 0.34 | 0.04 | 0.10 | 0.21 | 0.08 |  | - | - | - | - | - |  |
| FeO | 9.89 | 17.5 | 9.32 | 11.2 | 2.99 |  | 15.3 | 33.8 | 27.8 | 25.0 | 21.3 | 6.00 |  | 0.52 | 0.40 | 0.98 | 0.49 | 0.52 | 0.16 |
| MnO | 0.24 | 0.34 | 0.23 | 0.25 | 0.05 |  | 0.22 | 0.38 | 0.27 | 0.28 | 0.25 | 0.05 |  | - | - | - | - | - |  |
| MgO | 20.1 | 12.8 | 14.4 | 16.2 | 2.91 |  | 45.0 | 27.8 | 31.7 | 36.3 | 39.1 | 5.50 |  | 0.42 | 0.39 | 0.87 | 0.38 | 0.43 | 0.16 |
| CaO | 11.6 | 13.5 | 18.7 | 15.0 | 3.28 |  | 0.33 | 0.55 | 1.51 | 0.52 | 0.52 | 0.29 |  | 18.7 | 19.2 | 19.1 | 18.5 | 19.0 | 0.25 |
| Na_2_O | 0.05 | 0.02 | 0.04 | 0.04 | 0.01 |  | bd | bd | bd | bd | bd |  |  | 0.51 | 0.24 | 0.25 | 0.48 | 0.36 | 0.08 |
| K_2_O | - | - | - | - |  |  | - | - | - | - | - |  |  | 0.02 | 0.03 | 0.02 | 0.05 | 0.03 | 0.02 |
| Total | 98.7 | 99.2 | 99.2 | 99.1 |  |  | 100.3 | 97.9 | 99.5 | 99.4 | 99.6 |  |  | 100.1 | 99.7 | 99.1 | 99.7 | 99.6 |  |
|  |  |  |  |  |  |  |  |  |  |  |  |  |  |  |  |  |  |  |  |
| Wo | 24.5 | 30.0 | 40.6 | 32.4 | 7.4 |  |  |  |  |  |  |  |  |  |  |  |  |  |  |
| En | 59.1 | 39.6 | 43.5 | 48.7 | 7.8 |  |  |  |  |  |  |  |  |  |  |  |  |  |  |
| Fs | 16.3 | 30.4 | 15.8 | 18.9 | 5.4 |  |  |  |  |  |  |  |  |  |  |  |  |  |  |
| Mg# | 78.4 | 56.6 | 73.4 | 72.0 | 7.4 |  | 84.0 | 59.4 | 67.0 | 72.1 | 76.2 | 7.9 |  |  |  |  |  |  |  |
| An |  |  |  |  |  |  |  |  |  |  |  |  |  | 95.2 | 97.6 | 97.6 | 95.2 | 96.5 | 0.83 |
| Ab |  |  |  |  |  |  |  |  |  |  |  |  |  | 4.7 | 2.2 | 2.3 | 4.5 | 3.3 | 0.78 |

**Table S3. Chemical compositions of apatite standards, determined with an electron microprobe and H manometry***

|  | Ap-18 | Ap-3 | Ap-5 |
| --- | --- | --- | --- |
| P_2_O_5_ | 40.3 (2) | 40.1 (4) | 40.8 (4) |
| SiO_2_ | 0.00 (0) | 0.35 (2) | 0.26 (2) |
| Ce_2_O_3_ | 0.05 (4) | 0.64 (4) | 0.58 (6) |
| Y_2_O_3_ | 0.01 (1) | 0.08 (3) | 0.04 (3) |
| MgO | 0.01 (1) | 0.01 (1) | 0.01 (1) |
| CaO | 55.0 (2) | 54.4 (2) | 54.6 (2) |
| MnO | 0.00 (0) | 0.01 (1) | 0.07 (2) |
| FeO | 0.01 (1) | 0.03 (2) | 0.07 (2) |
| Na_2_O | 0.25 (2) | 0.26 (5) | 0.09 (1) |
| H_2_O^*^ | 0.20 (2) | 0.03 (1) | 0.37 (2) |
| F | 3.28 (19) | 3.44 (11) | 2.45 (11) |
| Cl | 0.13 (2) | 0.45 (6) | 0.95 (3) |
| S | 0.36 (3) | 0.13 (1) | 0.04 (1) |
| -O = F | 1.39 | 1.70 | 1.11 |
| -O = Cl | 0.03 | 0.10 | 0.21 |
| Total | 98.2 | 98.2 | 99.0 |

*Francis M. McCubbin did the analysis at the University of New Mexico, Albuquerque. Please refer to ref 19 for analytical method.

Numbers in parentheses are uncertainties (1σ) in the last digit of each value.

Ap-18, -3, and -5 are terrestrial apatite standards from Russia, Durango, and Colorado, respectively.
